# Evaluation of the Use of Parboiled Rice Hull Mulch for Weed Control in Outdoor Ornamental-Plant Production Environments

**DOI:** 10.3390/plants15091415

**Published:** 2026-05-06

**Authors:** Yuvraj Khamare, Stephen Christopher Marble

**Affiliations:** Mid-Florida Research and Education Center, Institute of Food and Agricultural Sciences, University of Florida, Apopka, FL 32703, USA; plantsages@gmail.com

**Keywords:** mulch, nursery crops, ornamental plants, pest management, rice hull

## Abstract

Parboiled rice hull mulch is becoming a widely used non-chemical weed control method in container nurseries, but research is lacking on the performance of rice hulls in outdoor production nursery environments. Two separate experiments were conducted to assess the effect of rice hull mulch on the emergence and growth of several common nursery weed species in an outdoor container nursery environment and to determine how well rice hulls performed over time. In a 12-week container experiment, rice hulls provided significantly better control of common container nursery weed species when seeds were placed on top of rice hull mulch applied at depths of 1.3 or 2.5 cm. In contrast, there was little difference in control when seeds were placed below the mulch layer regardless of mulch depth. In a long-term evaluation over 12 months, rice hulls applied at depths of 1.3, 2.5 or 5.0 cm provided control similar to pre-emergence herbicides through 6 months, but herbicides provided better control thereafter. Overall, rice hulls proved to be a suitable alternative to commercial herbicides and would be recommended in cases where pre-emergence herbicides cannot be used due to crop safety or grower preferences.

## 1. Introduction

Weeds are among the most problematic pests in container nursery production due to their competitive interactions for light, nutrients, water, and space, which can significantly reduce crop growth, yield, and marketability. Because of the confined growing environment within containers, weed competition can reduce container-grown crop growth by more than 60%, often resulting in extended production times [1,2]. Despite the severity of weeds, the container nursery industry has relatively few herbicide options that can be safely applied in or around ornamental crops [3]. This limitation is further compounded by the wide diversity of taxa produced in container nurseries, including succulents, herbaceous annuals, perennials, ornamental grasses, and tropical plants, many of which exhibit high sensitivity to herbicides. Additionally, increasing labor and chemical costs, concerns over herbicide runoff and leaching, consumer awareness of potential human health risks, and a growing emphasis on sustainable production practices have collectively encouraged growers to seek alternative weed management strategies [4,5,6]. As a result, non-chemical approaches, such as the use of organic mulches, have been increasingly adopted in container-grown plant production [4,7,8].

To date, most of the previous work on the use of mulch as a weed management method has focused on wood-derived products such as bark or wood chips [4,7,8]. However, a widely used material in container plant production is parboiled rice hulls [9]. Parboiled rice hulls are a byproduct of the rice milling process and are available at relatively low cost in large quantities from rice-producing states in the United States, including California, Louisiana, Mississippi, Arkansas, and Texas [10]. Rice hulls are well suited to use as a mulch in container-grown plants due to their hydrophobic nature, low bulk density, and ease of handling, shipping, and application [10]. In addition, rice hulls are commercially available and are currently used as components of greenhouse and nursery substrates as well as surface-applied mulch materials.

Previous research has demonstrated the effectiveness of rice hull mulch for weed control in container plant production [4,7]. Earlier research shows that rice hull mulch applied at depths of 1.3 and 2.5 cm provided complete control of flexuous bittercress (*Cardamine flexuosa*) and liverwort (*Marchantia polymorpha*) as indicated by having no weed presence in pots at these mulch levels [11]. Similarly, Altland et al. [12] found that rice hull mulch applied at depths of 1.3 to 2.5 cm significantly reduced the emergence and growth of flexuous bittercress and creeping woodsorrel (*Oxalis corniculata*), reducing shoot weight of these species by 75% to over 95% in comparison with a non-mulched control over a 16 wk study period. More recently, Khamare et al. demonstrated that rice hull mulch applied at depths of 1.3, 2.5, or 5 cm significantly suppressed the growth of flexuous bittercress, creeping woodsorrel, eclipta (*Eclipta prostrata*), longstalked phyllanthus (*Phyllanthus tenellus*), and liverwort at both early (1–2 leaf) and more advanced (2–4 leaf) growth stages [13], resulting in reductions of 56% to 100% in shoot dry weight across all species evaluated. Several studies suggest that the limited water retention capacity of rice hulls is a primary mechanism contributing to weed suppression by reducing moisture availability at the substrate surface [11,12].

Although research has clearly demonstrated the potential of rice hull mulch as a weed management tool in container production [7,8,11,12,13], these studies have primarily been conducted under greenhouse conditions and have evaluated a limited range of weed species, focusing primarily on *Oxalis* spp. and *Cardamine* spp. No or limited efficacy studies have been conducted on other important weed species such as spotted spurge (*Euphorbia maculata*), crabgrass (*Digitaria* spp.), doveweed (*Murdannia nudiflora*), or eclipta (*Eclipta prostrata*), which are regularly ranked as the most common or troublesome weeds in outdoor production of container-grown ornamentals [14]. If rice hulls are to be recommended as a primary non-chemical weed control strategy, data is needed for key weed species that are problematic outdoors, as many of these species have been omitted from previous investigations. Research is needed to evaluate the effectiveness of rice hull mulch in outdoor nursery production, focusing on its durability and its effects on controlling common weed species in outdoor settings. Therefore, two experiments were conducted to more closely assess the efficacy of rice hulls in outdoor production environments. First, a short-term study was initiated to determine the effect of rice hull depth and seed placement on the control of five common weed species. A second 12-month study was conducted to determine the long-term efficacy and durability of rice hulls over time in comparison with standard pre-emergence herbicide applications.

## 2. Results

### 2.1. Effects of Weed Seed Placement on Rice Hull Efficacy in Short-Term Container Experiments

For all five weed species evaluated, visual coverage ratings showed that weed growth was generally lower in pots in which the weed seeds were sown on top of the rice hull mulch layer, especially at the 2.5 cm depth (Table 1 and Table 2). For large crabgrass seeds placed below the mulch layer, weed coverage was similar or greater in pots mulched at 1.3 or 2.5 cm and non-mulched pots on all assessment dates in both experimental runs 1 and 2. Weed coverage was lowest in pots with large crabgrass seed placed on top of the mulch layer on most evaluation dates in experiment run 1, but fewer differences were noted in experiment 2. By 12 WAT in experiment 1, the highest crabgrass coverage was observed in pots with crabgrass seeds placed below the mulch layer, while the lowest coverage ratings were seen in pots with seeds placed above the mulch layer. With regard to shoot dry weight, the highest dry weights were recorded in pots with seeds placed below the mulch, while there was no difference in dry weight between the non-mulched control and pots with seeds placed on top of the mulch layer. While fewer differences were noted in weed coverage in experiment run 2, shoot dry weight data showed the best control was achieved with seeds placed on top of rice hulls applied at a 2.5 cm depth, with no differences in dry weights being observed between any of the other treatments and the non-mulched control group.

Results for eclipta were similar to crabgrass results overall. Rice hulls provided little to no benefit compared to the non-mulched control group on most evaluation dates when eclipta seeds were sown below the mulch layer. However, both the 1.3 and 2.5 cm rice hull mulch depths significantly reduced eclipta coverage when seeds were sown above the rice hull mulch, with the 2.5 cm depth providing the best control in experimental run 1, but with no difference in depth being observed in experimental run 2. With respect to eclipta shoot weight reduction, the 2.5 cm depth was the most effective treatment when seeds were sown above the rice hull mulch, while rice hulls provided no benefit when seeds were sown below the mulch. This trend was further observed in spotted spurge, where consistently lower coverage ratings and shoot dry weight were observed when seeds were sown above the mulch layer across both experimental runs.

In experimental run 1, the highest doveweed coverage was observed in pots with seeds sown below the rice hull mulch, which were higher than the non-mulched control group. The lowest coverage was consistently observed in pots with seeds sown above the mulch layer. Shoot dry weights showed the same general trend, with the exception that there was no significant difference in doveweed dry weight between the non-mulched control and the pots with seeds sown on top of the mulch. The highest shoot dry weight was again observed in pots with seeds sown below the mulch. In experimental run 2, the lowest coverage ratings were observed when seeds were sown above the mulch on most evaluation dates, but, differing from experimental run 1, some reductions were noted in pots with seeds sown below the rice hull mulch at the 2.5 cm depth in comparison with the non-mulched control. Dry weights again revealed the lowest shoot weight in pots with seeds sown on top of the mulch, but placing seeds beneath a 2.5 cm mulch depth provided a similar suppression in experimental run 2.

Similar to the other four weed species, the best longstalk phyllanthus control was achieved when seeds were placed on top of the mulch, especially at the higher 2.5 cm depth. In experimental run 1, pots with seeds placed below the mulch had similar or higher longstalk phyllanthus coverage as the non-mulched control. However, in experimental run 2, the treatments with seeds placed beneath the mulch layer provided some benefit, especially at the 1.3 cm depth, which also slightly suppressed shoot dry weight.

### 2.2. Long-Term Performance of Rice Hulls in Outdoor Production Environments

On all evaluation dates, both crape myrtles and loropetalum had similar growth indices in herbicide-treated pots and in rice hull mulch treatments, regardless of mulch depth or herbicide active ingredient. Few differences were noted among the various weed control treatments, especially at early evaluation dates. Regardless of herbicide or rice hull depth, weed coverage was similar among almost all treatments for the first 8 months of the experiment (Table 3). Exceptions did occur, however, specifically at 2 MAT in which weed growth was higher in crape myrtles treated with indaziflam compared to the pots treated with rice hull mulch (at any depth) or dimethenamid-P + pendimethalin, and in loropetalum in which pots treated with prodiamine + isoxaben had higher weed coverage than pots mulched at 2.5 or 5 cm.

While no differences were noted in the weed coverage ratings in either ornamental species at 4, 6, or 8 MAT, at 10 MAT, the lowest weed coverage in crape myrtles was observed in pots treated with indaziflam, while the highest weed coverage was observed in pots treated with rice hulls at 1.3 or 2.5 cm depths. While herbicide treatments generally provided the lowest weed coverage ratings in loropetalum at 10 MAT, results differed from crape myrtles in that the highest weed coverage was observed in pots mulched at the highest 5 cm mulch depth. At 12 MAT, dimethenamid-P + pendimethalin and indaziflam had the lowest weed coverage in crape myrtles, and had weed coverage similar to the prodiamine + isoxaben herbicide treatment and the 5 cm rice hull mulch depth. Fewer differences were noted in loropetalum at this time, with the only treatment difference being that prodiamine + isoxaben provided better weed suppression compared with the 2.5 cm mulch depth.

Weed shoot weights recorded at each 2-month period followed the same trend as weed coverage ratings, but fewer statistically significant differences were observed, mostly due to smaller weeds contributing to higher coverage ratings but being inconsequential to total shoot weights. In crape myrtles, the lowest shoot weights tended to be in pots that received herbicide, regardless of active ingredient, compared to rice hulls, especially at later evaluation dates beginning at 8 MAT (Table 4). This same trend was observed in loropetalum, albeit all treatments performed similarly in this ornamental species at the 10 MAT evaluation period.

Cumulative (total) shoot weed weight recorded in crape myrtle showed that across all 12 months of the experiment, the use of pre-emergence herbicides outperformed rice hulls at the 1.3 cm depth, and performed similarly to rice hulls when applied at 2.5 or 5 cm. Pre-emergence herbicides also resulted in the lowest total shoot weed weight in loropetalum; however, the 1.3 cm depth provided similar results to these herbicides, while the highest weed growth was observed in pots mulched at 2.5 and 5 cm depths. The reason higher weed growth was observed in pots with the highest depths of rice hulls is unclear, but follows the same trend where higher weed coverage ratings were seen in loropetalum mulched at the two highest depths.

**Figure 1:**
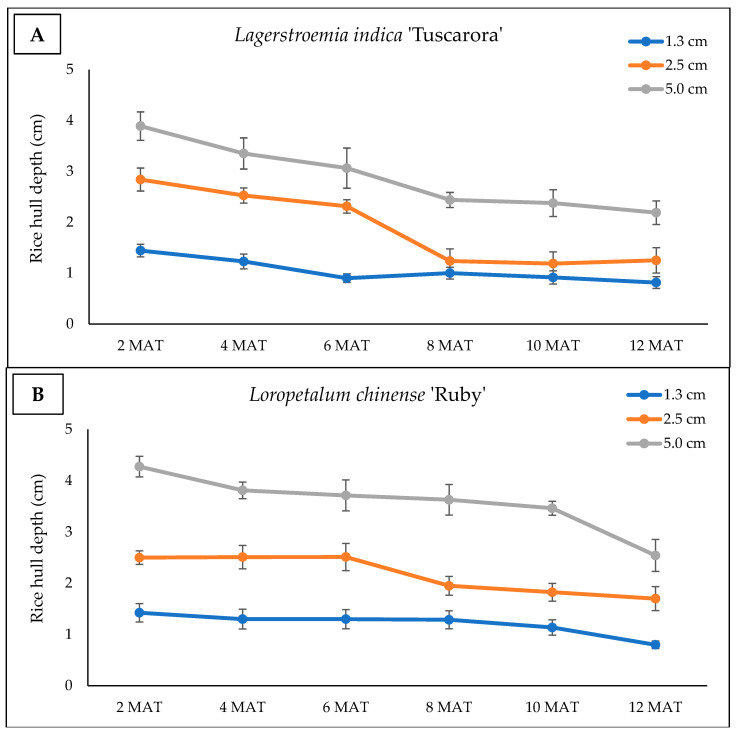
Parboiled rice hull mulch degradation under outdoor container nursery conditions following rice hull mulch application to container-grown (**A**) crape myrtle (*Lagerstroemia indica* ‘Muskogee’) and (**B**) loropetalum (*Loropetalum* chinense ‘Ruby’). Rice hull depths were measured bi-monthly at 2, 4, 6, 8, 10, and 12 months after mulch treatments were applied (MAT) at three different levels including initial depths of 1.3, 2.5, or 5 cm. Means and standard errors are shown.

Rice hull depth decreased in all treatments, with declines being observed most noticeably in the crape myrtle pots at 8 MAT when measuring the two highest initial depths of 2.5 and 5 cm. This aligns with both higher weed coverage ratings and higher weed shoot weights being observed in these containers at the 10 and 12 MAT evaluation periods, and greater control being achieved with pre-emergence herbicides. These herbicides were reapplied every two months and not subjected to the same degradation as a mulch would be which was applied only at study initiation (Figure 1).

## 3. Discussion

**Figure 2:**
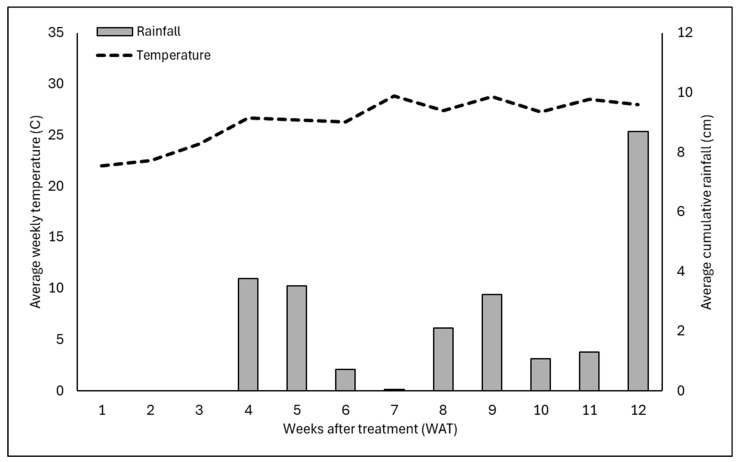
Average weekly temperature (C) and weekly cumulative rainfall (cm) over the 12-week study period for experimental run 1 of the short-term efficacy experiment. Experimental run 1 was initiated on 12 April 2024 and concluded at 12 weeks after initiation. Averages for each of the 12 weeks of the study are shown (WAT). Weather data were generated using the Florida Automated Weather Network (FAWN) weather stations located on the Mid-Florida Research and Education Center Campus in Apopka, FL, USA. (http://fawn.ifas.ufl.edu/; accessed on 16 April 2026).

**Figure 3:**
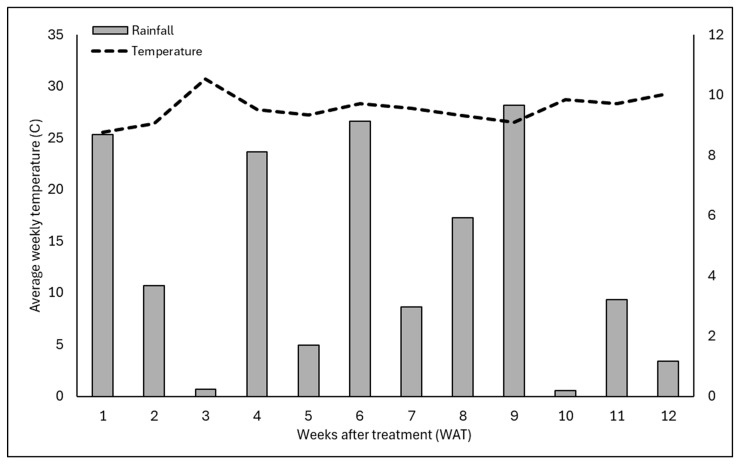
Average weekly temperature (C) and weekly cumulative rainfall (cm) over the 12-week study period for experimental run 2 of the short-term efficacy experiment. Experimental run 2 was initiated on 5 May 2025 and concluded at 12 weeks after initiation. Averages for each of the 12 weeks of the study are shown (WAT). Weather data were generated using the Florida Automated Weather Network (FAWN) weather stations located on the Mid-Florida Research and Education Center Campus in Apopka, FL, USA. (http://fawn.ifas.ufl.edu/; accessed on 16 April 2026).

In the short-term efficacy experiment, weed seed placement above the rice hull layer consistently provided a higher level of weed suppression compared to placement below the mulch layer, especially for seeds placed on top of 2.5 cm of mulch. Rice hulls have been consistently shown to provide a high level of weed control when seeds are placed on top of the rice hull mulch [8] due to the hydrophobic nature of rice hulls. In this study, pots were irrigated 1.3 cm per day while also receiving regular additional rainfall throughout the experiments (Figure 2 and Figure 3). Despite ample moisture being present and ideal temperatures for all of the weed species evaluated, weed growth was limited for these pots.

Research reported by Altland et al. [12] showed that rice hulls allowed for 88% to 96% of the applied irrigation or rainfall to pass through the mulch layer, and retain significantly less water than pine bark or peat moss substrates. In that study, rice hulls had a volumetric water content of less than 0.10 cm·cm^−1^ after 24 h following 1.3 cm of irrigation, compared to 0.4 cm·cm^−1^ for pine bark and peat, which are more conducive to weed growth [12]. Previous research has shown that optimal germination for spotted spurge occurs at 0 to −4 MPa (osmotic potential), with authors concluding that germination is likely limited to moist soils [15]. Similarly, significant reductions in germination have been noted for large crabgrass [16], *Phyllanthus* spp. [17], and doveweed [18]. While monitoring moisture levels within the rice hull mulch was beyond the scope of this experiment, it is still likely that moisture limitation was the primary mechanism leading to reduced emergence in this study. This mechanism of control would be in contrast to other mulch materials (bark, wood-derived products, etc.) that have been evaluated under similar nursery conditions, which are generally more effective on seeds placed beneath the mulch layer. With these materials, weed control is typically much higher when seeds are placed beneath the mulch layer, as these materials pose a greater physical barrier to emergence due to their weight, and allow for germination to occur within the mulch layer because they tend to retain more moisture following rainfall or irrigation events [19,20]. In contrast, rice hulls are lightweight by comparison, and while some reductions in emergence may be noted, they are most effective due to their ability to reduce moisture levels [11,12].

Rice hull mulch had little to no weed-suppressive effect when seeds were placed below the mulch layer for any of the five weed species evaluated. In fact, weed growth was often greater or similar to the non-mulched control when seeds were placed below the mulch, especially in experimental run 1. The reason for this is unclear but may be due to the lower rainfall that occurred in experimental run 1 vs. experimental run 2 (Figure 2 and Figure 3). While rice hulls have been shown to rapidly dry following rainfall or irrigation [12], research has shown that many different types of mulch increase moisture retention within the soil while slowing evaporation due to reductions in soil temperatures [21]. While continuous monitoring of soil moisture was beyond the scope of these studies, the presence of mulch could have created higher moisture levels within the mulched containers in comparison to the non-mulched control group. As the seeds that were placed beneath the rice hull mulch had access to this moisture, this could have led to increased growth.

Previous work has shown that rice hull depths of at least 1 cm can eliminate over 99% of photosynthetically active radiation as measured with a spectroradiometer placed beneath rice hull mulch applied at depths ranging from 0 to 2.5 cm [12]. For most of the species evaluated, germination is extremely reduced or inhibited in darkness including *Euphorbia* spp. [22], *Phyllanthus* species [17], doveweed [18], and eclipta [23]. In contrast, many *Digitaria* species can germinate in complete darkness [24]. While *PAR* was not recorded in this experiment, sufficient depths (over 1 cm) were applied that would theoretically exclude all, or almost all, of the *PAR* beneath the rice hull layer based on previous findings [12]. However, as this experiment was conducted outdoors, wind, water, and other disturbances likely altered the rice hull coverage, allowing for light transmittance and germination of weed seeds beneath the rice hull layer. Thus, even at the 2.5 cm depth, positively photoblastic seeds were able to readily germinate.

As light reduction and the presence of a physical barrier likely contributed little significance to weed control, it should also be mentioned that rice hull extracts have been shown to have allelopathic properties which can reduce the germination and growth of certain weed species such as barnyardgrass (*Echniochloa crusgalli*) [25,26]. However, allelopathic properties from rice products are typically extracts or other derivatives from raw, not parboiled, rice or rice hulls, which were utilized in this study [27]. While the allelopathic potential of parboiled rice hulls has not been investigated, the parboiling process would likely remove or significantly reduce the presence of any efficacious compounds that are found in fresh rice or rice byproducts. Further, parboiled rice hulls are widely used as a component of growing media in the nursery industry when incorporated into potting substrates, and thus would likely have no allelopathic potential for weed suppression [28].

Overall, data from this short-term evaluation of rice hull efficacy clearly demonstrates that rice hulls are most effective when weed seeds are placed on top of the mulch layer. Across all five species evaluated, weed coverage and shoot weights were lower in pots in which the seeds were placed above the rice hull mulch, and little to no benefits were realized from a weed control perspective when seeds were beneath the rice hull mulch. This aligns with previous findings by Altland et al. in which growth of *Cardamine flexuosa* and *Oxalis corniculata* was reduced when seeds were placed on top of rice hull mulch due to rapid drying and low volumetric water content within the mulch layer following rainfall or irrigation [12].

Results of the long-term study show that similar to other findings, rice hull mulch has limited or no impact on the growth of containerized woody ornamentals when applied at depths of 5 cm or less [11]. At least for the two ornamental species evaluated, rice hulls would provide no benefit in terms of plant growth in relation to a standard pre-emergence herbicide rotation, which echoes sentiments of previous research suggesting the added cost of mulch is more beneficial in species that are more herbicide-sensitive [13].

Rice hull depth decreased by approximately 40% to 60% over the course of the 12-month study. As only depth was measured, it is unknown if the reduction in rice hulls in each container was due strictly to degradation/breakdown, wind dispersal, or some other means. Research has shown that rice hulls have a relatively slow degradation rate compared to some other organic materials as they primarily consist of lignin, cutin, and insoluble silica [29]. When used as a container substrate component, previous work has shown rice hulls remained relatively stable with minimal degradation over a 70 wk. container study [29], but there were no previous long-term studies evaluating the stability of rice when used as a mulch. While this study examined rice hull depth reductions as they relate to weed management, further research is needed to examine degradation rates under different environmental conditions.

**Figure 4:**
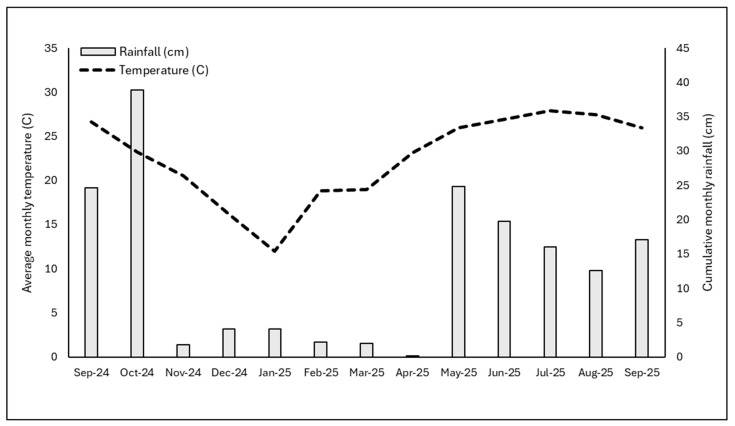
Average monthly temperature (C) and cumulative monthly rainfall (cm) over the 12-month long-term rice hull efficacy experiment. Weather data were generated using the Florida Automated Weather Network (FAWN) weather stations located on the Mid-Florida Research and Education Center Campus in Apopka, FL, USA (http://fawn.ifas.ufl.edu/; accessed on 16 April 2026). The experiment was initiated in September 2024 and concluded in September 2025.

While rice hull degradation occurred, the rice hull mulch still provided control similar to standard pre-emergence herbicide applications for the first 6 to 8 months of the experiment, indicating that they are a viable alternative to pre-emergence herbicides for short-term crops that reach a marketable growth stage within 6 to 8 months, or for herbicide-sensitive plants for which no herbicide options are available. While rice hull mulch depth consistently decreased in crape myrtles, depths were largely consistent in loropetalum pots through 6 MAT. Rice hull degradation mechanisms have not been extensively researched, but in addition to typical degradation and microbial breakdown, rice hulls are also prone to being blown out of containers by wind, especially when dry as they are lightweight compared to other mulch materials such as pine bark [29]. The upright growth habit of the crape myrtles likely caused rice hulls to be more prone to blowing out of the pots compared with the more low-growing and shrub-like loropetalum, which has a more dense plant canopy and theoretically would provide more protection from wind. Further, the more pronounced reduction in rice hull depth followed a 5-month period with low rainfall, which likely resulted in rice hulls containing less moisture during the day, and presumably more prone to wind dispersal out of the pots (Figure 4).

By 12 MAT, rice hull depths decreased by 39% to 56% in the crape myrtle and 38% to 49% in the loropetalum pots, averaging an approximate 50% decrease in mulch depth over the 12-month period. Changes in physical properties of rice hulls have been noted previously in long-term studies, with authors noting reductions in total porosity and bulk density and increases in water holding capacity, which could theoretically impact the weed suppressive ability of rice hulls over time [29]. However, previous long-term evaluations of rice hulls in nursery environments have focused on their use as a potting substrate amendment; thus, additional research is needed to more closely examine rice hull degradation and changes in physical and chemical properties over time when utilized as a mulch placed on top of the container surface.

## 4. Materials and Methods

### 4.1. Short-Term Efficacy of Rice Hulls in Response to Weed Seed Placement

Experiments were conducted at Mid-Florida Research and Education Center, Apopka, FL, USA, over a 12-week period in 2024 and repeated in 2025. On 12 April 2024, square nursery containers [1.68 L, 13.3 cm height, 13.3 cm diameter, Belden Plastics, St. Paul, MN, USA] were filled with a substrate consisting of pine bark, peat, and sand (80:10:10 *v*:*v*:*v*) and contained a previously incorporated controlled-release fertilizer [Osmocote^®^ Plus micronutrients 21-4-8 N-P-K (8–9 mo), ICL Specialty Fertilizers, Dublin, OH, USA] at a rate of 4.7 kg m^−3^. After filling pots, they were placed on a full-sun nursery container production pad and received 1.3 cm of overhead irrigation to settle the soil. Pots used to evaluate weed growth below a rice hull mulch layer were then seeded with 30 seeds (per pot) of either *Digitaria sanguinalis* (large crabgrass), *Eclipta prostrata* (eclipta), *Euphorbia maculata* (spotted spurge), *Murdannia nudiflora* (doveweed) or *Phyllanthus tenellus* (longstalk phyllanthus), with seeds of each species being sown onto separate groups of pots. All mulched pots were then mulched with parboiled rice hulls (Riceland Foods, Inc., Stuttgart, AK, USA) at depths of 1.3 or 2.5 cm while a control group received no mulch application. Depths of 1.3 and 2.5 cm were chosen as they are commonly used in commercial nursery production. Following mulch application, pots used to evaluate the efficacy of rice hull mulch with seeds placed on top of the mulch were seeded with 30 seeds of one of the five species in the same manner as described above. This yielded 5 experimental treatments consisting of pots that had weed seeds placed above or below a rice hull mulch layer at a 1.3 or 2.5 cm depth, with the addition of a non-mulched control group. All the containers were placed on a full-sun nursery pad, and weed species were grouped separately by species. Plants were irrigated 1.3 cm per day with overhead irrigation (Xcel-Wobbler sprinklers; Senninger Irrigation, Clermont, FL, USA) via two irrigation cycles (7:00 a.m. and 2:45 p.m.) throughout the experiment.

Data collection consisted of visual weed coverage ratings based upon a 0 to 100% scale where 0 = 0% of the container media surface covered with weed growth (or 100% control) and 100% (100% of the media surface covered with weed growth) every two weeks for 12 weeks after treatments (WAT) were applied. At 12 WAT, weed shoot growth was assessed by clipping weeds at the soil line and placing shoot tissues in a forced-air oven until a constant weight was reached.

The experiment was a completely randomized design with eight single-plant replications for each treatment and repeated on 5 May 2025 using the same methodology described above. Data were subjected to analysis of variance (ANOVA) using software (JMP Pro version 19; SAS Institute, Cary, NC, USA). Before the analysis, all data were inspected to ensure that the assumptions of the ANOVA were met. Data was grouped by year (i.e., experimental run) due to differences in climatic conditions that occurred in experimental run 1 vs. experimental run 2. Post hoc means comparisons were performed using Tukey’s honestly significant difference test, with differences considered significant at *p* = 0.05. Weather data were recorded using the Florida Automated Weather Network (fawn.ifas.ufl.edu) weather station located on site at the Mid-Florida Research and Education Center in Apopka, FL, USA. Mean average daily temperature and cumulative rainfall over each of the 12 weeks of the experimental period are reported in Figure 2 (experimental run 1) and Figure 3 (experimental run 2).

### 4.2. Long-Term Efficacy of Rice Hulls in Outdoor Production Environments in Comparison with Pre-Emergence Herbicides

This experiment was also initiated at the Mid-Florida Research and Education Center in Apopka, FL, USA, on 5 September 2024. Uniform, 5 cm fully rooted liners of crape myrtle (*Lagerstroemia indica* ‘Tuscarora’) and loropetalum (*Loropetalum chinense* ‘Ruby’) were obtained from local suppliers and transplanted into 11.3 L nursery containers [Nursery Supplies Inc., Kissimmee, FL, USA] using the same soil and amendments described previously. These two plant species were chosen in order to evaluate the efficacy and degradation of rice hulls when used to mulch an upright-growing ornamental (crape myrtle) alongside a shorter, more densely growing shrub (loropetalum) to account for different canopy structures of ornamental plants. Further, these two species are very commonly grown in Florida and throughout the southeastern United States.

Following potting, all plants were placed on a full-sun nursery pad and irrigated 1.3 cm per day with overhead irrigation via two irrigation cycles throughout the trial. On the day after potting, selected containers received an application of rice hull mulch described above at depths of 1.3, 2.5, or 5 cm. A separate group of plants were included and received no mulch, but were treated with one of three different granular pre-emergence herbicides including isoxaben + prodiamine (0.36 + 0.23 kg ai ha^−1^) (Gemini^®^ G, Everiss NA Inc., Dublin, OH, USA), dimethenamid-P + pendimethalin (1.68 + 2.24 kg ai ha^−1^) (FreeHand^®^, (BASF Corp., Research Triangle Park, NC, USA) or indaziflam (0.02 kg ai ha^−1^) (Marengo^®^ G, Envu Environmental Science U.S., LLC, Cary, NC, USA). Each herbicide was applied at standard manufacturer label rates every 8 weeks, similarly to a typical application schedule at commercial nursery operations. Due to the longer-term nature of the study, and the fact that natural weed pressure was sufficiently high, pots were not inoculated with specific weed seeds. Prior to application, all pots were weeded by hand to remove any emerged weeds and weed shoot fresh weight was recorded using a field balance every 2 months.

All data were recorded once every 2 months over the 12-month experimental period. Data collection included rice hull degradation, visual weed coverage ratings, weed shoot weight, and ornamental plant growth. Rice hull degradation was assessed by measuring the depth of rice hulls in each pot with a ruler, taking three separate measurements per pot, and then recording the average as an indication of the degradation of the rice hulls over time. Visual weed coverage ratings, weed fresh shoot weight, and weather conditions throughout the experimental period were measured as previously described. Daily average temperatures and cumulative rainfall are reported by month over the 12-month study period (Figure 4). To determine if treatments had any influence on crop growth, the growth index was assessed on crape myrtles and loropetalum plants by measuring plant height and two perpendicular width measurements. The trial was a completely randomized design with six single-plant replications for each mulch and herbicide treatment in each ornamental species. Loropetalum and crape myrtle data were assessed separately, with statistical analysis being conducted using the previously described procedures.

## 5. Conclusions

Overall, results from these experiments demonstrate that rice hulls are an effective weed management tool for outdoor container nursery production. As they were significantly more effective on seeds placed on top of the mulch, it would be recommended that growers apply the rice hull mulch as soon as possible after potting to prevent seed introduction prior to mulch application. Rice hulls also provided control similar to that of a standard pre-emergence herbicide rotation through 6 months but provided less control than pre-emergence herbicides at later evaluation dates, indicating some degradation or other losses occurred (i.e., wind).

While this study is the first report of the efficacy of rice hulls in an outdoor environment for certain key weed species, data is lacking on additional problematic weed species and this should be the focus of future research. As only depth measurements were taken, additional work is also needed to more closely examine rice hull degradation rates as they relate to different environmental and production conditions, as many factors such as irrigation frequency or temperature could affect results. Additionally, only two ornamentals were evaluated in this study from a growth perspective. Further work is needed to assess the impact of rice hull mulch on a wider variety of ornamentals, as well as to more fully understand how different rice hull mulch depths impact container moisture levels and temperatures over the course of a production cycle.

## Figures and Tables

**Table 1:** Experimental run 1: The effects of rice hull mulch depth and seed placement on the control of five common container nursery weed species over a 12-week period.

		Weed Coverage (%) ^1^			Weed Shoot Dry wt. (g) ^3^	Weed Coverage (%)			Weed Shoot Dry wt. (g)
Mulch Depth (cm) ^4^	Seed Placement ^5^	4 WAT ^2^	8 WAT	12 WAT		4 WAT	8 WAT	12 WAT	
		*Digitaria sanguinalis*				*Eclipta prostrata*			
1.3	Above	5 B ^6^	29 C	30 C	8.8 B	0 B	13 C	21 B	2.0 CD
2.5	Above	6 B	13 C	20 D	7.7 B	0 B	4 D	8 C	0.5 D
1.3	Below	75 A	83 A	90 B	22.5 A	31 A	49 AB	54 A	5.5 B
2.5	Below	73 A	81 A	100 A	24.7 A	24 A	51 A	43 A	9.9 A
0.0	NA	15 B	53 B	89 B	11.3 B	5 B	35 B	55 A	4.9 BC
		*Euphorbia maculata*				*Murdannia nudiflora*			
1.3	Above	0 B	16 C	20 D	1.8 C	1 B	15 C	35 C	1.9 B
2.5	Above	1 B	14 C	30 C	3.3 C	1 B	18 C	44 C	5.9 B
1.3	Below	81 A	76 A	90 B	6.4 B	23 A	72 A	100 A	14.2 A
2.5	Below	81 A	76 A	100 A	11.2 A	27 A	80 A	100 A	16.0 A
0.0	NA	20 B	44 B	100 A	6.3 B	0 B	47 B	90 B	4.4 B
		*Phyllanthus tenellus*							
1.3	Above	0 C	8 C	16 C	1.9 CD				
2.5	Above	0 C	1 C	6 D	0.5 D				
1.3	Below	13 A	48 A	44 B	4.9 AB				
2.5	Below	10 A	38 AB	60 A	5.3 A				
0.0	NA	3 B	21 B	70 A	3.4 BC				

^1^ Weed coverage was assessed visually on a scale of 0 to 100% where 0 = 0% of the surface of the container substrate covered with weeds and 100 = 100% of the surface of the container media covered with weeds. ^2^ WAT = weeks after treatments (rice hull mulch) were applied. Experiments were initiated on 12 April 2024. ^3^ Shoot dry weights were recorded by clipping weeds at the soil line and drying shoot tissues until a constant weight was reached. ^4^ Mulch depth shows the depth of parboiled rice hull mulch that was applied to pots. ^5^ Seed placement refers to the placement of 30 seeds/pot of each weed species relative to the rice hull mulch. NA = not applicable in reference to the non-mulched control group. ^6^ Means within a column followed by the same letter are not significantly different (Tukey’s HSD, *p* = 0.05).

**Table 2:** Experimental run 2: The effects of rice hull mulch depth and seed placement on the control of five common container nursery weed species over a 12-week period.

		Weed Coverage (%) ^1^			Weed Shoot Dry wt. (g) ^3^	Weed Coverage (%)			Weed Shoot Dry wt. (g)
Mulch Depth (cm) ^4^	Seed Placement ^5^	4 WAT ^2^	8 WAT	12 WAT		4 WAT	8 WAT	12 WAT	
		*Digitaria sanguinalis*				*Eclipta prostrata*			
1.3	Above	6 B ^6^	14 B	19 AB	9.0 AB	24 C	24 B	13 B	13.5 BC
2.5	Above	0 B	0 B	0 B	0.0 B	14 C	21 B	9 B	4.1 C
1.3	Below	6 B	28 AB	40 AB	16.2 AB	74 B	90 A	100 A	21.0 AB
2.5	Below	21 AB	36 AB	44 AB	23.9 A	61 B	90 A	100 A	25.6 A
0.0	NA	38 A	60 A	63 A	27.4 A	96 A	100 A	100 A	25.3 A
		*Euphorbia maculata*				*Murdannia nudiflora*			
1.3	Above	11 C	25 C	29 B	9.9 BC	9 C	45 BC	55 B	14.3 B
2.5	Above	6 C	19 C	24 B	8.1 C	2 C	13 C	21 B	5.9 B
1.3	Below	60 B	88 AB	90 A	20.1 A	20 B	83 AB	100 A	30.6 A
2.5	Below	48 B	63 B	85 A	16.0 AB	21 B	34 C	59 B	12.6 B
0.0	NA	100 A	99 A	100 A	17.1 A	53 A	94 A	100 A	36.0 A
		*Phyllanthus tenellus*							
1.3	Above	13 C	34 C	36 CD	16.5 C				
2.5	Above	5 C	10 D	16 D	12.1 C				
1.3	Below	31 B	59 BC	60 BC	17.9 BC				
2.5	Below	34 B	65 B	76 B	23.4 AB				
0.0	NA	100 A	99 A	100 A	24.4 A				

^1^ Weed coverage was assessed visually on a scale of 0 to 100% where 0 = 0% of the surface of the container substrate covered with weeds and 100 = 100% of the surface of the container media covered with weeds. ^2^ WAT = weeks after treatments (rice hull mulch) were applied. Experiments were initiated on 5 May 2025. ^3^ Shoot dry weights were recorded by clipping weeds at the soil line and drying shoot tissues until a constant weight was reached. ^4^ Mulch depth shows the depth of parboiled rice hull mulch that was applied to pots. ^5^ Seed placement refers to the placement of 30 seeds/pot of each weed species relative to the rice hull mulch. NA = not applicable in reference to the non-mulched control group. ^6^ Means within a column followed by the same letter are not significantly different (Tukey’s HSD, *p* = 0.05).

**Table 3:** Weed coverage ratings following treatment with either rice hull mulch or selected pre-emergence herbicides over a 12-month production cycle for two container-grown ornamental plant species.

			Mean Weed Coverage (%)					
Mulch Depth (cm) ^2^	Herbicide	Rate (kg ai ha^−1^)	2 MAT ^1^	4 MAT	6 MAT	8 MAT	10 MAT	12 MAT
			*Lagerstroemia indica* ‘Muskogee’					
1.3	None	0.0	4 B ^3^	1 A	7 A	15 A	39 A	41 A
2.5	None	0.0	4 B	1 A	2 A	13 A	31 AB	38 A
5.0	None	0.0	2 B	0 A	4 A	13 A	24 ABC	26 AB
None	Prodiamine + Isoxaben	0.36 + 0.23	11 AB	0 A	3 A	3 A	17 BC	21 AB
None	Dimethenamid-P + Pendimethalin	1.68 + 2.24	6 B	1 A	1 A	8 A	16 BC	3 B
None	Indaziflam	0.02	17 A	0 A	0 A	0 A	6 C	3 B
		*Loropetalum chinense* ‘Ruby’						
1.3	None	0.0	3 AB	0 A	1 A	3 A	3 B	13 AB
2.5	None	0.0	2 B	0 A	4 A	8 A	8 AB	35 A
5.0	None	0.0	0 B	0 A	1 A	21 A	28 A	25 AB
None	Prodiamine + Isoxaben	0.36 + 0.23	9 A	1 A	1 A	0 A	1 B	3 B
None	Dimethenamid-P + Pendimethalin	1.68 + 2.24	6 AB	0 A	0 A	0 A	3 B	5 AB
None	Indaziflam	0.02	6 AB	0 A	3 A	1 A	0 B	7 AB

^1^ MAT = months after treatment. The experiment was initiated in Apopka, FL, USA, in September 2024 and concluded in September 2025. ^2^ Mulch depth shows the depth of parboiled rice hull mulch that was applied to selected pots at the beginning of the experiment. ^3^ Means within columns followed by the same letter are not significantly different (Tukey’s HSD, *p* = 0.05).

**Table 4:** Weed shoot fresh wt. (g) following treatment with either rice hull mulch or selected pre-emergence herbicides over a 12-month production cycle for two container-grown ornamental plant species.

			Mean Weed Shoot wt. (g)						
Mulch Depth (cm) ^3^	Herbicide	Rate (kg ai ha^−1^)	2 MAT ^1^	4 MAT	6 MAT	8 MAT	10 MAT	12 MAT	Total ^2^
			*Lagerstroemia indica* ‘Muskogee’						
1.3	None	0.0	0.6 B ^4^	0.1 A	2.0 A	6.0 A	8.7 A	5.1 A	22.5 A
2.5	None	0.0	1.6 AB	0.1 A	0.3 AB	2.6 AB	7.0 AB	3.5 AB	15.1 AB
5.0	None	0.0	0.7 B	0.0 A	1.2 AB	2.8 AB	4.3 AB	2.7 B	11.6 B
None	Prodiamine + Isoxaben	0.36 + 0.23	1.8 AB	0.0 A	1.1 AB	0.6 B	4.4 AB	1.6 BC	9.5 B
None	Dimethenamid-P + Pendimethalin	1.68 + 2.24	1.0 B	0.0 A	0.0 B	2.4 AB	3.0 B	0.3 C	6.7 B
None	Indaziflam	0.02	6.4 A	0.1 A	0.0 B	0.2 B	3.4 B	0.5 C	10.5 B
		*Loropetalum chinense* ‘Ruby’							
1.3	None	0.0	0.0 B	0.0 A	0.1 A	1.0 AB	1.3 A	1.8 B	4.2 B
2.5	None	0.0	0.0 B	0.0 A	0.9 A	3.4 AB	3.9 A	8.7 A	16.9 A
5.0	None	0.0	0.0 B	0.0 A	0.6 A	3.8 A	3.6 A	8.1 A	16.1 A
None	Prodiamine + Isoxaben	0.36 + 0.23	1.0 AB	0.5 A	0.4 A	0.5 AB	0.0 A	0.0 B	2.4 B
None	Dimethenamid-P + Pendimethalin	1.68 + 2.24	1.4 A	0.0 A	0.0 A	0.2 B	0.0 A	0.0 B	1.6 B
None	Indaziflam	0.02	0.1 B	0.0 A	0.8 A	0.3 B	0.0 A	0.0 B	1.2 B

^1^ MAT = months after treatment. The experiment was initiated in Apopka, FL, USA in September 2024 and concluded in September 2025. ^2^ Total shows cumulative weed biomass recorded in each treatment over the completed 12 mo. experiment. ^3^ Mulch depth shows the depth of parboiled rice hull mulch that was applied to selected pots at the beginning of the experiment. ^4^ Means within columns followed by the same letter are not significantly different (Tukey’s HSD, *p* = 0.05).

## Data Availability

The raw data supporting the conclusions of this article will be made available by the authors on request.
